# Drought risk in the Anthropocene

**DOI:** 10.1098/rsta.2021.0297

**Published:** 2022-12-12

**Authors:** Jim W. Hall, Jamie Hannaford, Gabi Hegerl

**Affiliations:** ^1^ Environmental Change Instittute, University of Oxford, UK; ^2^ UK Centre for Ecology & Hydrology, Wallingford, Oxon, UK; ^3^ School of Geosciences, University of Edinburgh, Edinburgh, UK

The summer of 2022 has seen remarkable hydrological conditions across much of the Northern Hemisphere. Europe has seen what could be the most severe drought conditions to hit the continent for more than 500 years [1], with exceptionally low water levels impacting navigation of inland waterways and leaving insufficient cooling water for power plants. French fire fighters struggled with wildfires on a dramatic scale. The Yangtze River in China reached historically low levels. In the Western USA, the drought has broken previous records for the driest 22-year period in the region since the year 800 CE [2], depriving farmers of water and further impacting fragile ecosystems in the Colorado River basin. Examining these various manifestations of drought reveals a series of interlinked phenomena that are examined in this themed issue of the *Philosophical Transactions of the Royal Society*.

First, it is clear that droughts are profoundly human-influenced, and we expect them to be even more so in the future. The title of this themed issue—*Drought risk in the Anthropocene*—reflects the recognition that the Earth System is human-influenced to an extent that we are said to be living in a new geological era [3]. Droughts are a manifestation of the Anthropocene in a number of different senses. As the paper by Vicente-Serano *et al*. (Global drought trends and future projections) [4] explains, greenhouse gas emissions are driving some of the conditions that are making droughts more severe. Human influences both warm the planet and intensify contrasts between wet and dry conditions, and drought has been impacted particularly by increased evaporation linked to warming. Human influences may also be implicated in circulation changes that are shifting the patterns of meteorological drought conditions. Marvel *et al*. (Using machine learning to identify novel hydroclimate states) [5] use a new combination of empirical data and machine learning methods to identify the increasing frequency of drought conditions in the twentieth and twenty-first centuries.

Besides anthropogenic climatic changes, human activity has impacted the Earth's surface and catchments through land use change, urbanization and infrastructure construction. These changes have modified the ways in which meteorological droughts propagate into hydrological droughts. The expansion of irrigated agriculture and water use in cities and industries means that human water withdrawals are often the most significant drivers of water scarcity. Since the introduction of the concept of planetary boundaries by Rockström *et al*. in [6], there has been debate about whether human exploitation of fresh water is now so voluminous that a planetary boundary for water has already been exceeded. Pastor *et al*. (Understanding the transgression of global and regional freshwater planetary boundaries) [7] continue their investigation of this question, by examining water stress at regional as well as global scales.

Second, the processes by which droughts materialize are extraordinarily complex. They vary enormously across space. Readers will obtain insights into the complexity of local processes by reading the basin-scale and regional analyses in this themed issue, including Mishra *et al*.'s article (Benchmark worst droughts during the summer monsoon in India) [8], which examines the severity of monsoon droughts, and Quentin Grafton *et al*.'s article (Resilience to Anthropogenic Drought in the Northern Murray-Darling Basin, Australia) [9], which adds to the important literature on the complex hydrology and contested water use in the Murray–Darling Basin. The paper by Murgatroyd *et al*. (Strategic analysis of the drought resilience of water supply systems) [10] illustrates the complexity of how meteorological droughts translate into hydrological droughts and water shortages for people. The interactions are influenced above all by human water management: storage, transfers and the allocation of water to different users. These papers illustrate the role of basin-scale and national-scale studies in understanding the complexity of droughts. Though there have been very impressive steps in global hydrology and water resource systems modelling over the last two decades, it has far from replaced the insights that can be gained from more local-scale studies that can mobilize much more data to expose the complexity of hydrological processes.

Finally, droughts have multiple impacts and those impacts are profoundly modified by adaptation responses. Quentin Grafton *et al*.'s article examines the response of birds in the Murray-Darling Basin, which adds to ongoing debates in the literature on the ecological impacts of droughts. Ecosystems are naturally resilient to droughts, but when they are already degraded because of other anthropogenic impacts, extreme droughts may push ecosystems into a state from which they cannot recover—more research is required to establish under what conditions that hypothesis is true. This emphasizes the point that climate change needs to be viewed in the context of multiple stressors on the environment.

Barbier's [11] review article on the economics of managing water crises examines the economic impacts of droughts and argues that misallocation of water is a consequence of inadequate pricing of scarce water resources. Few economists would argue with that assertion, though they may also point to the very rare instances of functioning water markets and question why the governance conditions to create such markets are so elusive.

This themed issue documents important scientific advances that are improving understandings of droughts and the evidence needed for drought management. There have been rapid advances in drought forecasting, which are documented in the paper by AghaKouchak *et al*. (Status and prospects for drought forecasting: opportunities in artificial intelligence and hybrid physical-statistical forecasting) [12]. These advances have been enabled by a combination of new data sources, in particular from satellite Earth observation, and increasingly powerful methods to analyse these vast datasets, notably with machine learning (also employed by Marvel *et al*. [5] for analysis of climate data) and artificial intelligence. AghaKouchak *et al*. [12] demonstrate how physical insights can help to constrain and improve the performance of data-based methods. Increasing computational resources are enabling climatic, hydrological and water resource systems modelling on scales that were hitherto considered to be infeasible—for example the paper by Murgatroyd *et al*. [10] applies water resource system simulation modelling, driven by more than 9000 years of synthetic daily climate data to analyse water supply shortages on an unprecedented national scale. The paper by Goodwin *et al*. (The contribution of a catchment-scale advice network to successful agricultural drought adaptation in Northern Thailand) [13] reports on lessons learnt from social network analysis, applied to data gathered from an extensive on-the-ground survey of farmers, in guiding ways forward for improved adaptation to foster improved agricultural drought resilience in the region.

As one would expect from the Chief Executive of England's Environment Agency (which has responsibility for regulating the water environment and drought planning), Sir James Bevan's concluding article (Drought risk in the Anthropocene: from the jaws of death to the waters of life) [14] focuses upon what can and should be done to manage drought risk. The article follows the speech by Sir James in 2019, which used the ‘jaws of death’ analogy to draw attention to the combined effects on future water shortages of increasing demand for water and climate-driven water scarcity. In his article for this themed issue, Sir James points out opportunities for a more drought-resilient future, which may be achieved through reductions in water consumption, strategic augmentations of supply and reallocating more water to the aquatic environment to build its resilience to climate-related shocks. Such an approach will need to be underpinned by a better scientific understanding of the causes and management of droughts, which this themed issue seeks to provide.

## Data Availability

This article has no additional data.
